# Spatiotemporal Imaging of Glutamate-Induced Biophotonic Activities and Transmission in Neural Circuits

**DOI:** 10.1371/journal.pone.0085643

**Published:** 2014-01-15

**Authors:** Rendong Tang, Jiapei Dai

**Affiliations:** 1 Wuhan Institute for Neuroscience and Neuroengineering, South-Central University for Nationalities, Wuhan, China; 2 College of Biomedical Engineering, South-Central University for Nationalities, Wuhan, China; 3 Department of Pharmacology, College of Pharmacy, South-Central University for Nationalities, Wuhan, China; University of British Columbia, Canada

## Abstract

The processing of neural information in neural circuits plays key roles in neural functions. Biophotons, also called ultra-weak photon emissions (UPE), may play potential roles in neural signal transmission, contributing to the understanding of the high functions of nervous system such as vision, learning and memory, cognition and consciousness. However, the experimental analysis of biophotonic activities (emissions) in neural circuits has been hampered due to technical limitations. Here by developing and optimizing an *in vitro* biophoton imaging method, we characterize the spatiotemporal biophotonic activities and transmission in mouse brain slices. We show that the long-lasting application of glutamate to coronal brain slices produces a gradual and significant increase of biophotonic activities and achieves the maximal effect within approximately 90 min, which then lasts for a relatively long time (>200 min). The initiation and/or maintenance of biophotonic activities by glutamate can be significantly blocked by oxygen and glucose deprivation, together with the application of a cytochrome c oxidase inhibitor (sodium azide), but only partly by an action potential inhibitor (TTX), an anesthetic (procaine), or the removal of intracellular and extracellular Ca^2+^. We also show that the detected biophotonic activities in the corpus callosum and thalamus in sagittal brain slices mostly originate from axons or axonal terminals of cortical projection neurons, and that the hyperphosphorylation of microtubule-associated protein tau leads to a significant decrease of biophotonic activities in these two areas. Furthermore, the application of glutamate in the hippocampal dentate gyrus results in increased biophotonic activities in its intrahippocampal projection areas. These results suggest that the glutamate-induced biophotonic activities reflect biophotonic transmission along the axons and in neural circuits, which may be a new mechanism for the processing of neural information.

## Introduction

The processing of neural information in neural circuits plays a key role in neural functions [1], [2] and functional neural circuits have been extensively investigated with *in vitro* and *in vivo* combinations of electrophysiological recording techniques and other approaches [3]–[5]. It is well accepted that neuronal communication is mediated by bioelectricity and chemical molecules via the processes called electrical and chemical transmission, respectively, which mainly occur in axons and synapses [6]. Indeed, they seem to provide explanations for the basic functions of the nervous system, however, the wide array of experimental observations regarding electrical and chemical transmission have made it difficult to construct general accepted concepts or principles to provide reasonable explanations of higher neural functions, such as sensory and motor control, vision, learning and memory, and cognition and consciousness. Therefore, many unanswered questions and debates over the neural encoding and mechanisms of neuronal networks remain [7]–[12]. For instance, the number of spikes fired by neurons that originate from electrical and chemical transmission have been considered to be the primary mechanism for the encoding of neural information (rate coding); however, the fire rate is not fully correlated to neural functions, and it is even very sparse or silent for most of the neurons in the hippocampus, neocortex and cerebellum under the appropriate behavioral conditions [8], [9].

Almost all life, including microorganisms, plants, animals and human beings, can spontaneously radiate extremely weak photon beam in the normal or pathological conditions. Such a phenomenon is known as biological ultra-weak photon emissions, or UPE (in short biophotons) [13]–[15]. Although the existence of this extremely weak biophotons was first reported in the 1920s [16], however it had not been verified until 1950s when the photomultiplier tube was invented [17]. After that, new experimental evidence has demonstrated the generation mechanism of biophotons, which is related to the mitochondrial respiration, lipid oxidation and other metabolic activities [18], [19]. In addition, it has been believed that biophotons, being a coherent electromagnetic field inside the cells, may be the base of cell-to-cell communication [20], which has been demonstrated in plants, bacteria and certain animal cells [21], [22].

It has been suggested that biophotons may play a potential role in neural signal transmission, contributing to the understanding of the high functions of nervous system [23]–[26]. More recently, biophotonic activities (emissions) have been found to conduct along neural fibers [23], which suggests that biophotonic activities may play an important role in the communication of neural signals and in the functioning of the nervous system. The experimental analysis of biophotonic activities and transmission in neural circuits, however, has been hampered by technical limitations.

## Results

### *In vitro* biophoton imaging method and glutamate-induced biophotonic activities in mouse coronal brain slices

**Figure 1:**
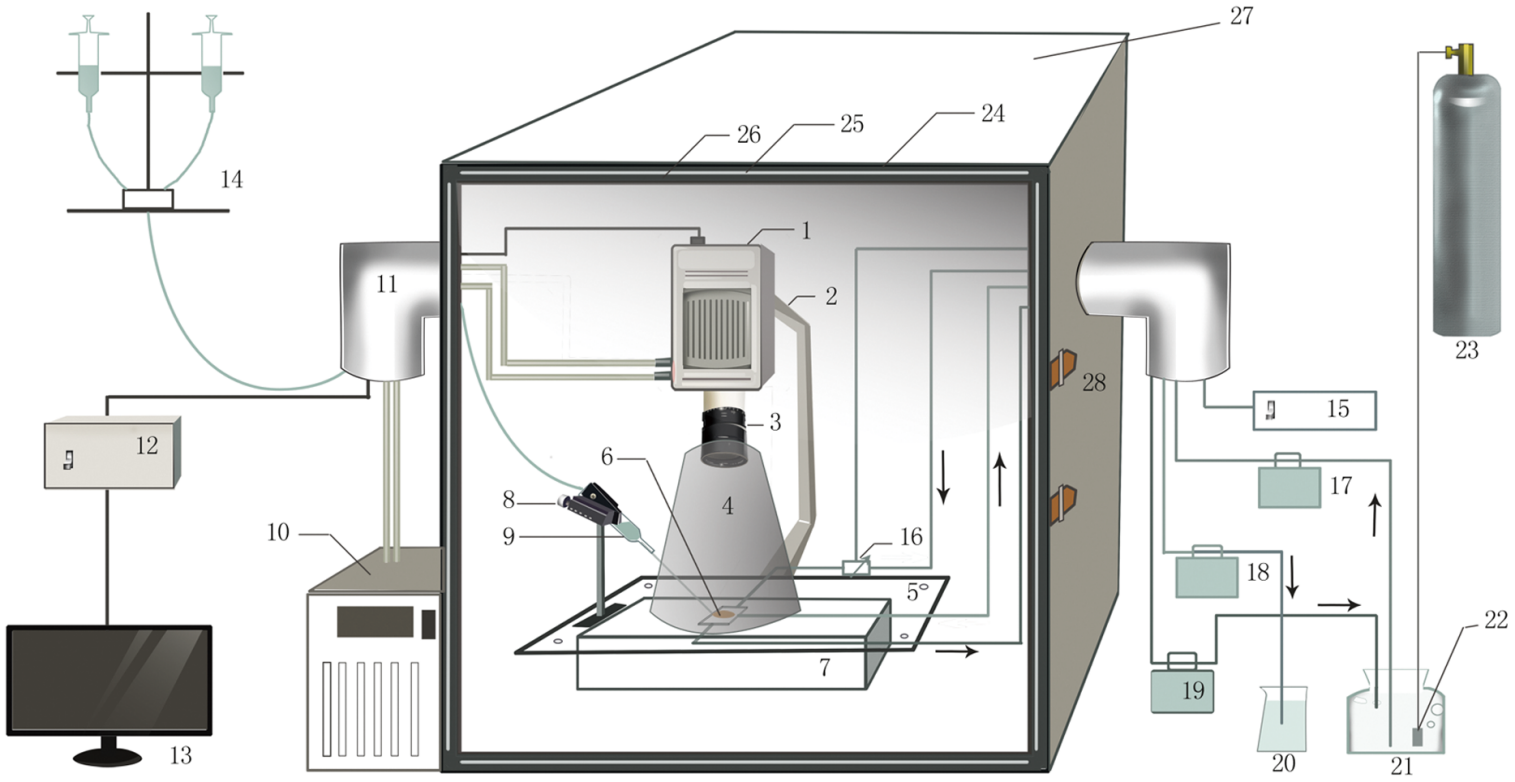
Schematic drawing of the biophoton imaging system. EM-CCD camera (1); EM-CCD camera supporter and up-down adjuster (2); Lens (3); Conical light isolation cover (black cotton cloth) for lens and sample (4); Sample stage (5); Perfusion chamber and sample (6); Sample stage supporter and adjuster (7); Micromanipulator (8); Pressure control perfusion system: solution delivery device (9); Cooling water circulation pump (10); Bent stainless steel tube (11); EM-CCD controller (12); Computer (13); Pressure control perfusion system (14); Electrical heating controller (15); Electrical heater (16); Input micropump for chamber perfusion (17); Output micropump for chamber perfusion (18); Output micropump for local perfusion (19); Collective beaker for local perfusion (20); Glass bottle for chamber perfusion medium (21); Membrane oxygenator (22); Gas cylinder (23); Ion layer of dark box (24); Lead plate layer of dark box (25); Black cotton cloth layer of dark box (26); Dark box (90 cm×70 cm×110 cm) (27).

**Figure 2:**
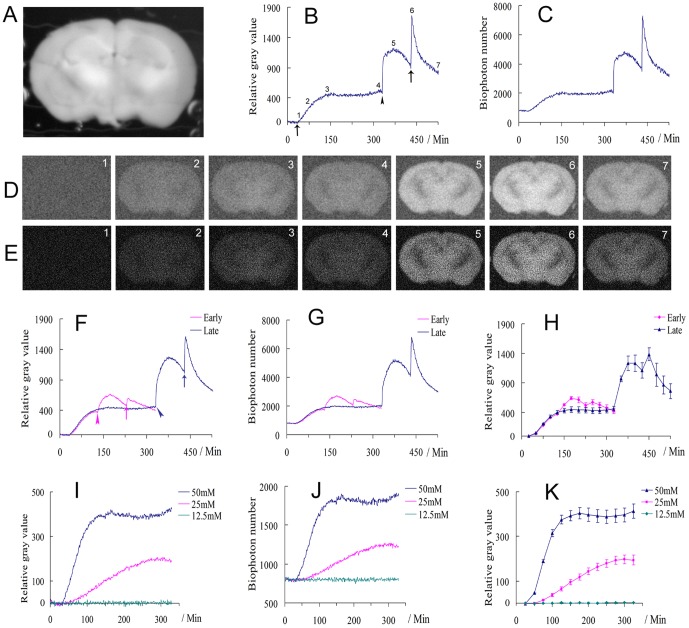
Imaging biophotonic activities (emissions) after the application of glutamate in mouse coronal brain slices. (**A–E**) A representative regular image of a coronal brain slice (**A**). The dynamic change of biophotonic activities in this slice was demonstrated by relative gray values (RGVs, **B**) and biophoton numbers (BPNs, **C**). Representative biophoton gray images (**D**) and corresponding biophoton number images (**E**) at the selected time periods indicated in **B** (digit:1–7) after the application of 50 mM glutamate. Each image in **D** or **E** was obtained from the merger of 25 continuously processed original gray images or biophoton number images (1 min imaging time for each original image, see also Figure S1). The time points are indicated in **B** for the first and second application of 50 mM glutamate (arrow) and slice washing (arrowhead). (**F, G**) The sum of the time course of the average change of RGVs (**F**) and BPNs (**G**) (blue line, n = 6), and the effects of early slice washing (pink arrowhead) and the second application of 50 mM glutamate (pink arrow) (pink line, n = 5) are much less relative to the late treatments (blue arrowhead and arrow, see also in **B**). (**H**) The sum of the time course of the average change of RGVs in **F** from 25 continuously processed original gray images. (**I–K**) Dose-dependent changes of biophotonic activities (**I** and **J**) and the sum of the time course of the average change of RGVs from the 25 continuously processed original gray images (**K**); no obvious effect was found at the concentrations of 12.5 mM (n = 4), the time to reach the maximal effect was longer, and the amplitude of the maximal effect was significantly less at 25 mM than that at a concentration of 50 mM (**K**, 232.3±7.4 versus 91.5±7.9 min; 211.4±22.4 versus 410.2±30.9 RGVs, p<0.001, n = 6 for 25 or 50 mM). 1 min imaging time for each time point in **B**, **C**, **F**, **G**, **I** and **J**. Data show mean±s.e.m. n = the number of slices from the same number of mice.

We developed and optimized an *in vitro* biophoton imaging system (Figure 1) by improving the efficacy of biophoton detection, eliminating the ambient light emission and performing additional imaging processing and analysis. Consequently, the biophotonic activities of the mouse brain slices bathed in a routine artificial cerebrospinal fluid (ACSF) were able to be detected and imaged spatiotemporally. However, the detected signals of biophotonic activities in mouse brain slices were still weak, so we investigated whether neuronal signaling molecules could stimulate or enhance biophotonic activities. Glutamate, the most abundant excitatory neurotransmitter in the nervous system, can produce neural activities such as long-term potentiation (LTP) and neural spikes in brain slices [27], [28]. Because the concentration of glutamate stored in the presynaptic vesicles is 60–200 mM [29], we tested the application of different concentrations of glutamate (12.5, 25 and 50 mM) to mouse coronal brain slices to observe their effect on biophotonic activities and obtained a basic and typical finding after the application of 50 mM glutamate, based on the analysis of the relative gray values (RGVs) and the biophoton numbers (BPNs) of the images (Figure 2; Figure S1 and S2). A long-lasting application of 50 mM glutamate led to a gradual and significant increase of biophotonic activities and achieved the maximal effect within approximately 90 min (91.5±7.9 min, n = 6, Figure 2B, 2F and 2H, called the effect of initiation), which remained stable for a relatively long time (>200 min, Figure 2B–H, the effect of maintenance). Interestingly, a lasting slice washing with ACSF after a 300-min application of glutamate led to a further increase of biophotonic activities within approximately 12.6 min (12.6±1.8 min, n = 6), which then decayed gradually (Figure 2B, 2C, 2F and 2G, washing effect). Surprisingly, the second application of 50 mM glutamate after slice washing for 100 min produced a quick increase of biophotonic activities and achieved a much higher response within approximately 3.5 min (3.5±0.2 min, n = 6, Figure 2B, 2C, 2F and 2G, reapplication effect). The effects of slice washing and the second application of 50 mM glutamate tended to be weaker if the slice washing began at the early period of the achievement of the maximal effect (Figure 2F–H). Therefore, the typical patterns of biophotonic activities by the application of 50 mM glutamate could be identified as four main periods or stages (initiation, maintenance, washing and reapplication, respectively).

### The factors influencing glutamate-induced biophotonic activities

**Figure 3:**
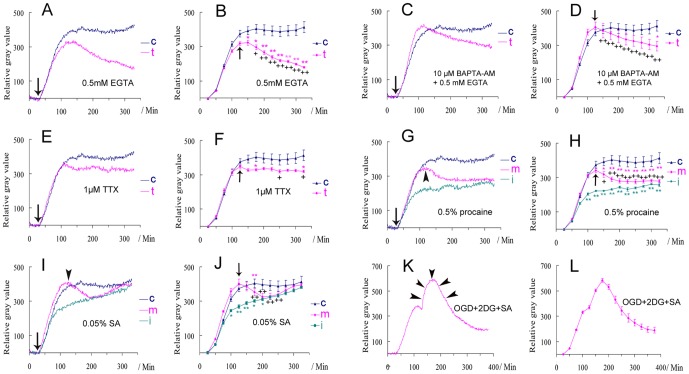
The effects of different treatments on the biophotonic activities in mouse coronal brain slices. (**A–D**) Removing extracellular Ca^2+^ (**A** and **B**, 0.5 mM EGTA, n = 6) or removing intra- and extracellular Ca^2+^ together (**C** and **D**, 10 µM BAPTA-AM+0.5 mM EGTA, n = 6) from the beginning of the application of 50 mM glutamate (pink line in **A** and **C**). The changes were significant during the maintenance period (**B** and **D**). (**E, F**) 1 µM TTX had no influence on the initiation, but partly on the maintenance (pink line, n = 5). (**G, H**) Both initiation (green line, n = 5) and maintenance (pink line, n = 6) were significantly affected by 0.5% procaine. (**I, J**) Both initiation (green line, n = 6) and maintenance (pink line, n = 6) were significantly affected by 0.05% sodium azide (SA), but a recovery was found after long-lasting application. Arrows indicate the treatment at the beginning of the application of 50 mM glutamate in **A**, **C**, **E**, **G** and **I** and arrowheads mark the treatment that began after the achievement of the maximum effect in **G** and **I**. **t:** treated group; **c:** control group (blue line) is same in **A–J** (n = 6), **i**: the treatment together with application of glutamate (initiating period), **m**: the treatment during the maintenance period. Data show mean±s.e.m. n = the number of slices from the same number of mice. * treated group (corresponding color) versus control at the same time periods; + the maximum effect just before treatment versus the effects after (arrows in **B**, **D**, **F**, **J**) in treated group. * or + P<0.05, ** or ++ P<0.01. (**K, L**) Oxygen and glucose deprivation (OGD, 95% N+5% CO2), together with the application of 5 mM 2-deoxy-D-glucose (2DG) and 0.05% SA during the maintenance period, tended to significantly inhibit the biophotonic activities in a gradual way (n = 6), but the effect of slice washing still existed and even lasted for a relatively long time (increasing part, arrowheads).

We then investigated how the 50 mM glutamate-induced biophotonic activities were influenced. Removing extracellular Ca^2+^ from the bathing medium (0.5 mM EGTA, Figure 3A and 3B), removing intracellular and extracellular Ca^2+^ together (10 µM BAPTA-AM and 0.5 mM EGTA; Figure 3C and 3D), or applying an action potential inhibitor (1 µM TTX; Figure 3E and 3F) at the beginning of the application of glutamate did not block the initiation of biophotonic activities (from the glutamate application to the achievement of maximal effect), but the maintenance of the maximal effect was affected in such situations (Figure 3A–F). Interestingly, both the initiation and maintenance of biophotonic activities were significantly affected by the application of a regional anesthetic (0.5% procaine) at the beginning of the application of glutamate or when the maximal effect was achieved (Figure 3G–H).

Furthermore, we studied whether the glutamate-induced biophotonic activities were dependent on energy metabolism and aerobic metabolic pathways. The initiation and/or maintenance of biophotonic activities was partly blocked by cytochrome c oxidase inhibitor (0.05% sodium azide, SA), but recovery was observed after the long-lasting application of SA (Figure 3I and 3J). The maintenance of biophotonic activities was blocked by oxygen glucose deprivation (OGD, 95% N+5% CO_2_) together with the application of 5 mM 2-deoxy-D-glucose (2DG) and 0.05% SA; however, this blocking effect was a gradual process (Figure 3K and 3L).

### Biophotonic activities and transmission in mouse sagittal brain slices

**Figure 4:**
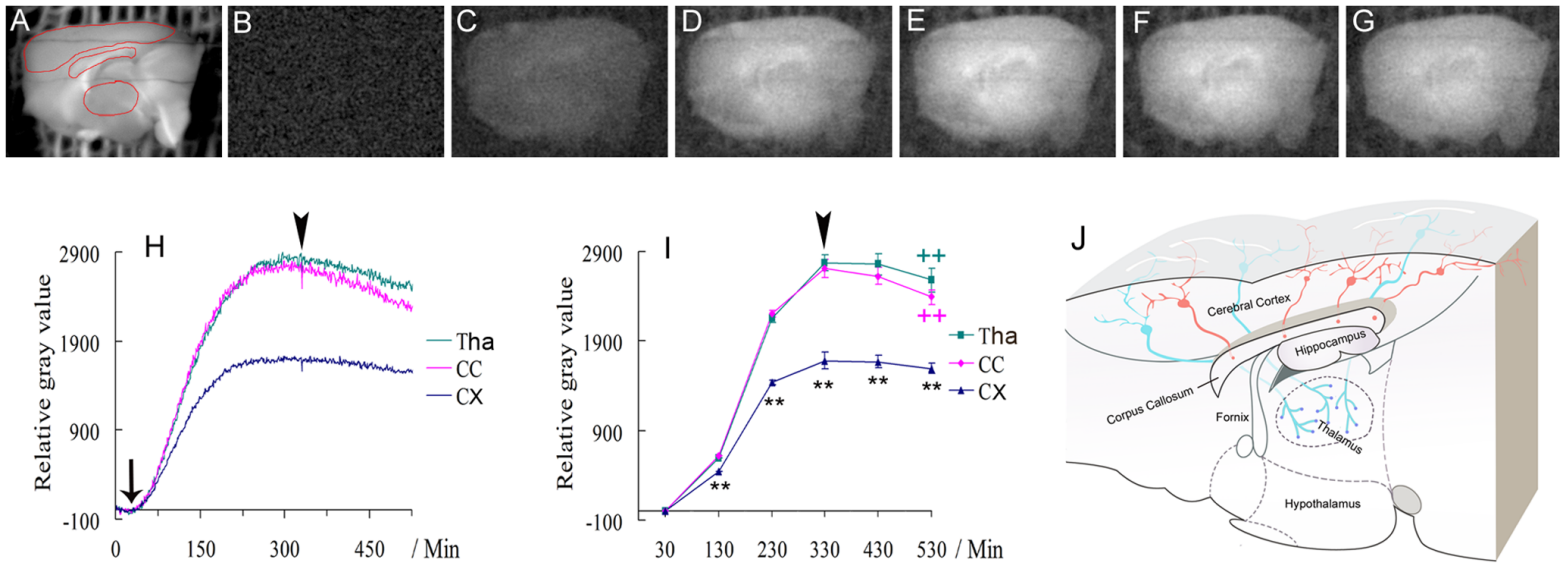
Imaging biophotonic activities and transmission in mouse sagittal brain slices. (**A**) A representative regular image of sagittal brain slices and the regions of interest (ROIs), including the cerebral cortex, corpus callosum and thalamus, are marked by red lines, which were selected for the analysis of biophotonic activities. (**B–I**) Representative biophoton gray images at the selected time periods before (**B**–**E**) and after (**F**, **G**) the application of Protein Phosphatase 2 inhibitor (OA). Each image was obtained from the merger of 100 continuously processed original grey images. The sum of the time course of the average change of RGVs (**H**, 1 min imaging time for each time point) and the sum of time course of average change of RGVs from the 100 continuously processed original gray images (**I**) in the cerebral cortex, corpus callosum and thalamus before and after the application of OA (n = 7 slices from the same number of mice). The significant increase of biophotonic activities in the corpus callosum and the thalamus (**C–E, H, I**) tended to decay after the application of OA (**F–I**). Arrow and arrowheads in **H** and **I** indicate the time points for the long-lasting application of 50 mM glutamate and 200 nM OA, respectively. Data show mean±s.e.m. **CX**: cerebral cortex; **CC**: corpus callosum; **Tha**: thalamus. * **CC** or **Tha** group (corresponding color) versus **CX** group at the same time periods; + the effects after application of OA versus the maximum effect (arrowhead) before in each group. * or + P<0.05, ** or ++ P<0.01. (**J**) A schematic explanation for the origin of biophotonic activities in the corpus callosum and thalamus in a sagittal brain slice. The cut end of axons in the corpus callosum (red spots) and the cut end of axonal terminals in the thalamus (blue spots) originate from the cortical projection neurons (red and green, respectively). Such anatomic projection patterns of cortical projection neurons indicate that the detected biophotonic activities in the corpus callosum and thalamus originate from the cortical projection neurons via the biophotonic transmission along their axons or axonal terminals.

In order to investigate the origin of glutamate-induced biophotonic activities with particular emphasis on whether they could transmit along neuronal axons or in neural circuits, and the possible transmission mechanisms, we first employed sagittal brain slices in which the cut end of the corpus callosum (white matter) faced to the lens of imaging system (Figure 4A) to observe the biophotonic activities after the long-lasting application of 50 mM glutamate. A schematic explanation for the detection and analysis of the origin of biophotonic activities and transmission by using such a novel experimental design is demonstrated in Figure 4J. Consequently, we found that the extensive biophotonic activities were located at the area of the corpus callosum and the thalamus (Figure 4C–E and 4H). We then observed the biophotonic activities upon the application of Protein Phosphatase 2A (PP2A) inhibitor (Okadaic acid potassium salt, OA) after 300 min of imaging (Figure 4F–I). The inhibition of PP2A activity can induce the hyperphosphorylation of microtubule-associated protein tau and interferer with the function of microtubules [30], while the microtubules or other proteins are theoretically considered to be possible components that mediate biophotonic or bioelectromagnetic communication along axons [23], [31], [32]. We found that the application of 200 nM OA significantly decreased the glutamate induced-biophotonic activities in the corpus callosum and the thalamus (Figure 4F–I).

### Biophotonic activities and transmission in the intrahippocampal circuits

**Figure 5:**
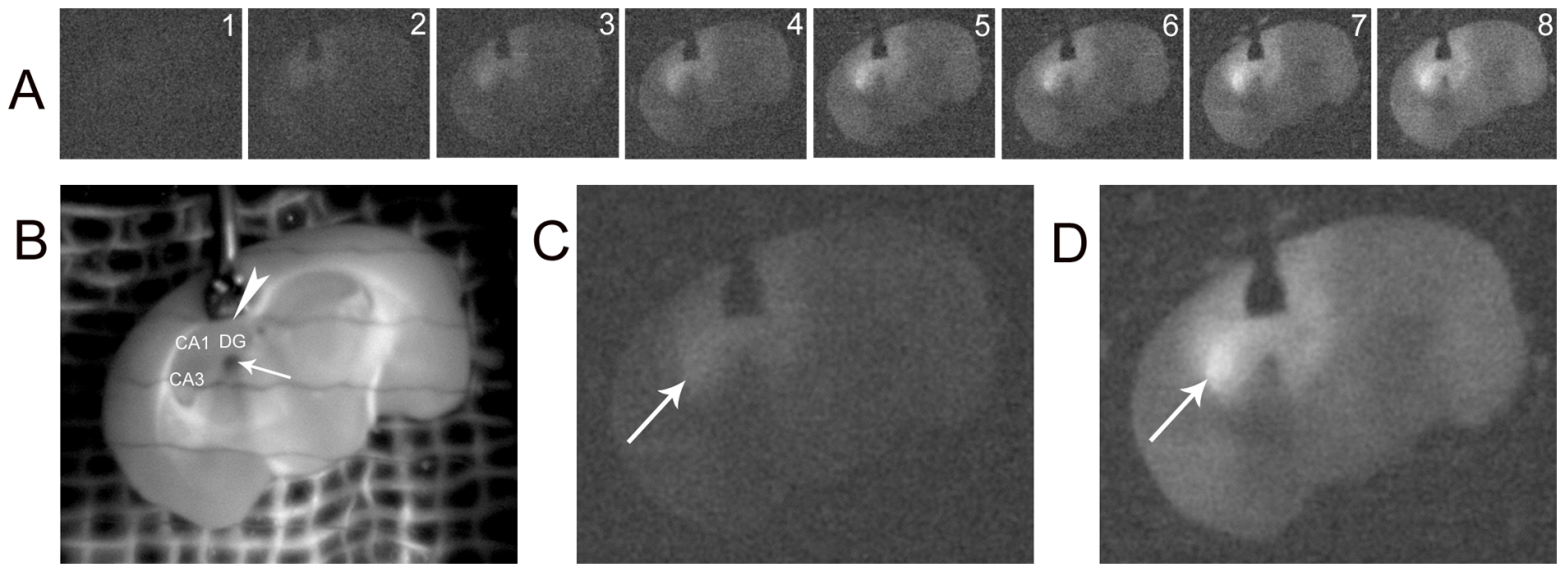
Biophotonic activities and transmission in intrahippocampal circuits. (**A**) The spatiotemporal change of biophotonic activities in a coronal brain slice at the selected time periods (digit:1–8) after the local application of 75 mM glutamate to hippocampal dentate gyrus (DG) together with whole slice perfusion with 25 mM glutamate. Each image was obtained from the merger of 25 continuously processed original gray images (1 min imaging time for each original image). (**B**) A regular image of this coronal brain slice showing the local perfusion of the hippocampal DG at the rostral part of the hippocampus. Arrow and arrowhead indicate the input and output perfusion sites, respectively, and DG, CA2 and CA1 areas in the hippocampal formation are shown. (**C, D**) Two enlarged biophoton gray images obtained from the merger of 100 continuously processed original gray images at the selected time periods (images **A**_1–4_ for **A** and **A**_5–8_ for **D**), showing an increase of biophotonic activities in the ipsilateral CA3 (arrow in **C** or **D**), indicating biophotonic activity transmission from DG to CA3. Such an observation was repeated in 5 slices from the same number of mice.

Finally, we observed the spatiotemporal pattern of biophotonic activities in the coronal brain slices with the additional application of glutamate in the hippocampal dentate gyrus via a local perfusion method. We carried out the long-lasting application of 75 mM glutamate to one side of dentate gyrus at the rostral part of hippocampus, together with a perfusion of 25 mM glutamate for the whole slice (Figure 2A and 2B). According to our primary observations, the perfusion of relatively low concentration of glutamate (25 mM) for the whole slice could maintain a basic level of biophotonic activity, and such a treatment could enhance the biophotonic activities in the projection areas of the dentate gyrus after an additional local perfusion of high concentration of glutamate (75 mM) to the dentate gyrus. Consequently, we found that the application of glutamate to the dentate gyrus led to an increase of biophotonic activities in the ipsilateral CA3 (Figure 5A, 5C and 5D), the well-identified intrahippocampal main projection area of the dentate gyrus, indicating the presence of biophotonic activities and transmission in the intrahippocampal circuit of the dentate gyrus after the application of glutamate.

## Discussion

In the present study, we identified the biophotonic activities and transmission in mouse coronal and sagittal brain slices by the application of very high concentration of glutamate (25–75 mM). Although such high concentrations of glutamate are still within the low level of the concentrations of glutamate stored in the presynaptic vesicles (60–200 mM) [29], however, an argument would be raised on whether the detected biophotonic activities and transmission along neuronal axons or in neural circuits reflect a physiological mechanism or just a response to pathophysiological conditions or even neuronal injuries caused by high concentrations of glutamate. It has been generally believed that extracellular exposure to a high concentration of glutamate would be toxic to neurons, called glutamate neurotoxicity, which has been suggested to contribute to neuronal death in brain injuries such as ischemia and trauma, and in other neurological disorders [33], [34]. However, increasing evidence suggests that glutamate neurotoxicity may vary greatly under different experimental conditions, and is influenced by different factors. For example, local, high extracellular concentrations of glutamate may be well tolerated in the intact brain, but not in situations where energy supply is limited (stroke and anoxia) or deficient (mitochondrial dysfunction) [35]. In a previous study [36], by using microdialysis probes incorporating an electrode to apply glutamate or glutamate-receptor agonists and measuring the amplitude of the resulting depolarization in the rat striatum *in vivo*, it was found that 10 to 100 mM glutamate (estimated EC50 = 42 mM) were needed to produce marked deflection of the local direct current potential. This argues against the oversimplified hypothesis that high extracellular glutamate is the key to neurotoxicity in the certain experimental conditions or in neurological disorders [35]. Therefore, the specific patterns of the biophotonic activities and transmission after application of high concentrations of glutamate under our experimental conditions do not certainly arise from the effect of glutamate neurotoxicity. Whether there exists any interaction effect between biophotonic activities and transmission and possible neurotoxicity after application of high concentrations of glutamate, however, needs to be investigated further.

In this study, we found that the initiation and/or maintenance of biophotonic activities can be significantly blocked by oxygen glucose deprivation together with the application of 2DG and cytochrome c oxidase inhibitor, but only partly by cytochrome c oxidase inhibitor, suggesting that the glutamate-induced biophotonic activities were dependent on energy metabolism and that alternative anaerobic metabolic pathways may be a compensatory method to meet energy requirements when the aerobic metabolic pathways are inhibited.

Previous studies on other types of cells have shown that the origin of biophotonic activities is mostly a result of the generation of various reactive oxygen species (ROS), the common products of aerobic metabolism (mitochondrial respiration) [37], [38]. Here, we found that the increase of biophotonic activities by the application of glutamate was not correlated to the change in aerobic metabolism because cytochrome c oxidase inhibitor did not completely block the initiation and maintenance of biophotonic activities. Therefore, our findings suggest that the glutamate-induced biophotonic activities may comprise the component of active biophotons that play a role in biophotonic transmission in neural cells and neural circuits, while aerobic metabolism may only be involved in the generation of background biophoton component, as proposed in our previous study [23].

To address our proposal, we observed the glutamate-induced biophotonic activities in mouse sagittal brain slices and found that the extensive biophotonic activities were located at the area of the corpus callosum and the thalamus, and were significantly decreased by the application of PP2A inhibitor (OA), suggesting that the extensive biophotonic activities in these two areas may mainly originate from axons or axonal terminals in the corpus callosum and thalamus, respectively, which may be at least partly due to the active biophotonic transmission along the axons or axonal terminals of cortical projection neurons (Figure 4J).

Interestingly, the initiation of biophotonic activities by glutamate is independent of the action potential and extracellular and intracellular Ca^2+^, but the maintenance of the maximal effect was influenced when the action potential was absent or when the extracellular and intracellular Ca^2+^ were removed constantly. However, both the initiation and maintenance of biophotonic activities were significantly affected by the application of a regional anesthetic (procaine), suggesting a new possible mechanism for anesthetics by interfering with biophotonic transmission via the inhibition of action potential and the function of microtubules [39]. These results illustrate biophotonic activities and transmission in relation to electrical and chemical transmission, and it seems that either electrical or chemical transmission may only provide a basis for the initiation and maintenance of biophotonic activities and transmission. Although our findings allow for the reconsideration of the traditional views on the roles of electrical and chemical transmission, which are traditionally believed to be important mechanisms for the processing of neural information including the neural encoding and mechanisms of neuronal networks [7]–[12], however, some key questions remain to be answered. For example, what is the mechanism of biophotonic transmission in neural circuits? How are biophotons involved in synaptic signal transmission? How do we construct novel models for neural information coding according to biophotonic activities and transmission, and should quantum theory be considered based on photon behavior characteristics [40]–[42]? The answers to these questions should help to explain the fundamental mechanisms of neural communications, and the functions of nervous system, such as vision, learning and memory, cognition and consciousness, and the mechanisms of human neurological and psychiatric diseases.

## Conclusions

Taken together, we have developed and optimized an *in vitro* biophoton imaging system, which can spatiotemporally demonstrate the biophotonic activities and transmission that may help to understand the mechanism for the processing of neural information. In addition, this new imaging method may have potential for wide use in other research fields.

## Materials and Methods

### Mouse brain slice preparation

Coronal and sagittal brain slices were obtained from male Kunming mice (2–3 months). This study was carried out in strict accordance with the recommendations in the Guide for the Care and Use of Laboratory Animals of the National Institutes of Health. The protocol was approved by the Committee on the Ethics of Animal Experiments of South-Center University for Nationalities. (Permit Number: AEP2010-004). All surgery was performed under sodium pentobarbital anesthesia, and all efforts were made to minimize suffering. Mice were decapitated, and the brain was quickly removed and placed in ice-cold (4°C) artificial cerebral spinal fluid (ACSF) for approximately 3 min. The thin coronal brain slices were cut to 450 µm thickness with a Vibratome (Leica VT1000S, Germany) beginning at the rostral part of hippocampus. The thick coronal brain slices were cut to 2-mm thickness beginning at the rostral part of hippocampus. For the preparation of a particular sagittal brain slice from a hemisphere, the whole brain was carefully separated into two hemispheres and the sagittal brain slice (2-mm thickness) with the medial surface of the corpus callosum was prepared from one hemisphere with a Vibratome. The slices were incubated in ACSF at room temperature (∼23°C) for a minimum of 1 hr before imaging. ACSF contained (in mM): 125 NaCl, 2.5 KCl, 2 CaCl_2_, 1 MgCl_2_, 1.25 NaH_2_PO_4_, 26 NaHCO_3_, and 10–20 D-glucose, pH 7.4, continuously bubbled with 95% O_2_ and 5% CO_2_.

### *In vitro* biophoton imaging system and imaging processes

The *in vitro* biophoton imaging system (Figure 1) mainly consists of a new generation of ultra low light detector-electron multiplier CCD camera (EM-CCD) (C9100-13, Hamamatsu Photonics K. K., Hamamatsu, Japan) mounted with a Navitar's high-speed fixed focal length lens (DO-5095, Navitar, USA) and a stereomicroscopic supporter, which are set in a complete dark box in dark room. The EM-CCD camera is fixed on the top of the stereomicroscopic supporter and can be moved up and down with a hand-manipulated adjuster. The EM-CCD camera is cooled by a cooling water circulation pump and controlled by a computer and image analysis software (HCImage Version 1.1.3.1, Hamamatsu Photonics K. K., Hamamatsu, Japan). A chamber is fixed on the sample stage, and a specific conical light isolation cover made from the black cotton cloth is attached to the lens. The cover is used to isolate the lens and sample chamber to maintain complete darkness in the imaging field. The electrical cables and the experimental pipes pass through two bent stainless steel tubes attached on the two sides of the dark box.

The slices were transferred to the chamber, where they were submerged beneath continuously superfusing ACSF (100 ml) that was contained in a glass bottle. A mixture of 95% O_2_+5% CO_2_ was constantly supplied with a membrane oxygenator placed in the ACSF during the perfusion period. The perfusion was maintained through an input micropump and an output micropump (∼5 ml/min) outside the dark box. The temperature (∼31°C) of the medium in the perfusion chamber was maintained with an electrical heater. The restricted application of glutamate in the hippocampal dentate gyrus was maintained by combining a pressure control perfusion system (MPS-2, WPI, USA) with an additional micropump (0.2 ml/min). Oxygen and glucose deprivation was achieved by removing glucose from ACSF and continuously bubbling with 95% N_2_+5% CO_2_ together with adding 5 mM 2-deoxy-D-glucose (2DG).

Biophotonic activities (emissions) were detected and imaged using the EM-CCD camera in water-cool mode (in this situation, the working temperature at the CCD camera can be maintained as low as −90°C). The other setup parameters for the EM-CCD camera during imaging were 1200×gain, 1×1 binning, and photon detection model 3. The specific steps for biophoton detection and imaging were as follows: (1) the slices were transferred to a chamber in complete darkness for approximately 30 min before imaging to exclude the effects of ambient light; (2) real-time imaging by automatically taking an image every 1 min; (3) a regular image was taken under the ambient light conditions before and after the imaging processes had been finished.

### Image processing and data analysis

The specific processes for image processing and data analysis were as follows: (1) all original images were processed with a program running at the MATLAB platform. This image processing method resulted in two kinds of images: the processed biophoton gray images and the biophoton number images; (2) eliminating the effect of cosmic rays from the original gray images (white spots, Figure S1A) resulted in the processed biophoton gray images (Figure S1B); (3) the biophoton number images (Figure S1C) were obtained from the processed gray images by an additional image processing program developed according to the parameters of EM-CCD camera and run on the MATLAB platform; (4) the processed images then were carried out for the analysis of the average gray values (AGVs) in the regions of interest (ROIs) with image analysis software (HCImage Version 1.1.3.1, Hamamatsu Photonics K. K., Hamamatsu, Japan), and the relative gray values (RGVs) were obtained according to a method described in Figure S2; (5) the biophoton numbers (BPNs) in ROIs were also obtained from the biophoton number images with the same image analysis software. It should be emphasized that the consecutive biophoton gray and number images were merged to generate an average image depending on the purpose of image analysis.

### Statistical analysis

Statistical analyses were performed using Microsoft Excel, and details of the results are described in Table S1.Two-tailed Student's *t*-test was used to compare the effects between the control and treated group at different time points and the two-tailed paired *t*-test was used to compare the effects at different time points in the treated groups.

### Drug preparation

Glutamate (12.5, 25, 50 and 75 mM, Sigma, St. Louis, MO, USA), procaine (0.5%, Sigma, Steinheim, Germany), sodium azide (0.05%, Sanland-chem International INC, Shanghai, China), 2-deoxy-D-glucose (5 mM, Sigma, St. Louis, MO, USA) and TTX (1 µM, More-Bio, Wuhan, China) were directly dissolved in the ACSF. BAPTA-AM (10 µM, Molecular Probes, Eugene, OR, USA), EGTA (0.5 mM, Amresco, Solon, OH, USA) and okadaic acid potassium salt (OA, 200 nM, LC Lab, Woburn, MA, USA) were initially dissolved in DMSO and then diluted to their final concentration in the ACSF.

## Supporting Information

Figure S1 **The images obtained from a representative slice with or without performing imaging processing.** (**A**) The images without performing imaging processing. (**B**) The images are used for the measurement of average gray values (AGVs) after performing imaging processing by eliminating the effect of cosmic rays. (**C**) The images are used for the measurement of biophoton numbers (BPNs) after transferring to biophotonic images. Each image was obtained from the merger of 25 continuous original images (the imaging time was 1 min for each original image).
(ZIP)

Figure S2 **An example of the analysis of the relative gray vales.** (**A–C**) A regular image of coronal brain slice (**A**) and two biophoton gray images before (**B**) and after the application of 50 mM glutamate (**C**). The image of the coronal brain slice is marked (red line, **A**) and the outlined area is superposed to other biophoton gray images (**B** and **C**) for the measurement of the average gray values (AGVs) in the region of interest (ROI: here, the whole slice) on each image. The image analysis program can return the AGVs for each traced ROI on each image, and therefore, the relative gray vales (RGVs) of the traced ROI of the image in **C** can be calculated and defined as: RGVs (**A**) = AGVs (**B**) - AGVs (**C**). Here, the image in **B** or **C** is an average gray image generated from the merger of 25 continuous original images. This method of analysis can be used for any original and merged images depending on the purpose of analysis. In addition, the background AGVs [here is AGVs (**B**)] used for the calculation of RGVs in a given image is chosen from a mean of average gray values from 30 continuous original images (30 min imaging time) before the application of glutamate to maintain accuracy.
(ZIP)

Table S1 **Statistical results.**
(DOC)
