# Supplementary figures and images for: Spatiotemporal Imaging of Glutamate-Induced Biophotonic Activities and Transmission in Neural Circuits
Source: PLoS One. 2014 Jan 15;9(1):e85643. doi: 10.1371/journal.pone.0085643 (PMC3893221; doi:10.1371/journal.pone.0085643)

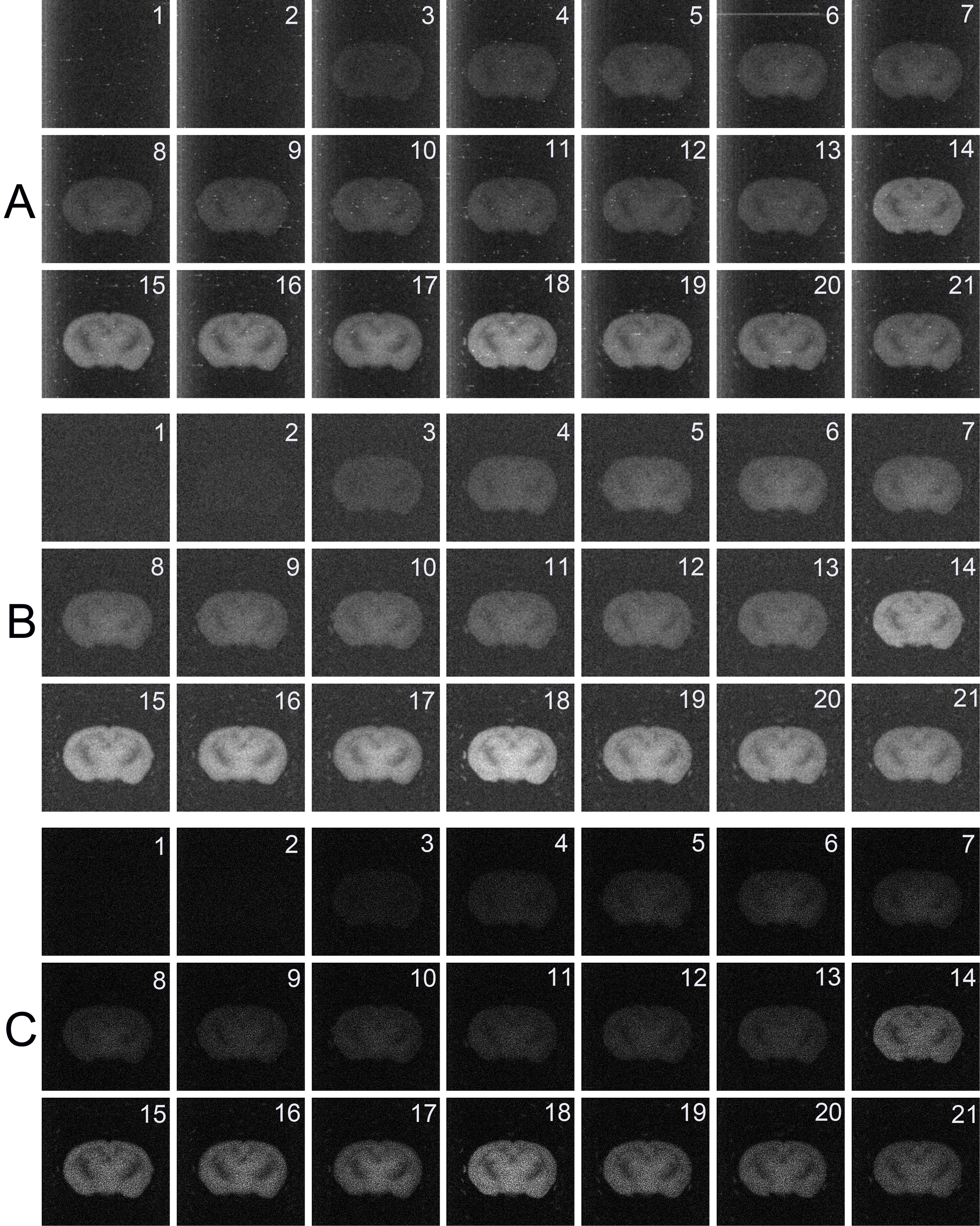

Supplement: Figure S1 — The images obtained from a representative slice with or without performing imaging processing. (A) The images without performing imaging processing. (B) The images are used for the measurement of average gray values (AGVs) after performing imaging processing by eliminating the effect of cosmic rays. (C) The images are used for the measurement of biophoton numbers (BPNs) after transferring to biophotonic images. Each image was obtained from the merger of 25 continuous original images (the imaging time was 1 min for each original image). (ZIP) [file pone.0085643.s001.zip › Figure S1.tif]

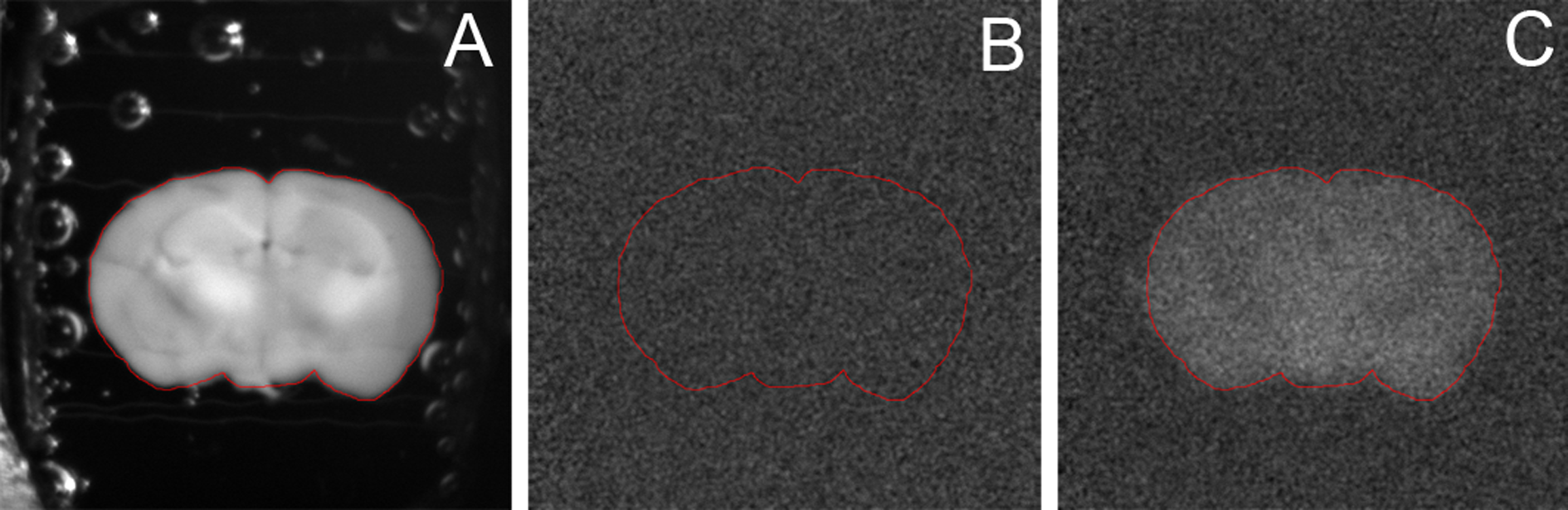

Supplement: Figure S2 — An example of the analysis of the relative gray vales. (A–C) A regular image of coronal brain slice (A) and two biophoton gray images before (B) and after the application of 50 mM glutamate (C). The image of the coronal brain slice is marked (red line, A) and the outlined area is superposed to other biophoton gray images (B and C) for the measurement of the average gray values (AGVs) in the region of interest (ROI: here, the whole slice) on each image. The image analysis program can return the AGVs for each traced ROI on each image, and therefore, the relative gray vales (RGVs) of the traced ROI of the image in C can be calculated and defined as: RGVs (A) = AGVs (B) - AGVs (C). Here, the image in B or C is an average gray image generated from the merger of 25 continuous original images. This method of analysis can be used for any original and merged images depending on the purpose of analysis. In addition, the background AGVs [here is AGVs (B)] used for the calculation of RGVs in a given image is chosen from a mean of average gray values from 30 continuous original images (30 min imaging time) before the application of glutamate to maintain accuracy. (ZIP) [file pone.0085643.s002.zip › Figure S2.tif]
