# Supplementary material for: Spatiotemporal Imaging of Glutamate-Induced Biophotonic Activities and Transmission in Neural Circuits
Source: PLoS One. 2014 Jan 15;9(1):e85643. doi: 10.1371/journal.pone.0085643 (PMC3893221; doi:10.1371/journal.pone.0085643)
Supplement: Table S1 — Statistical results. (DOC) [file pone.0085643.s003.doc]

| **Table S1. Statistical results** | | | | |
| --- | --- | --- | --- | --- |
| Fig. 2 |  |  |  |  |
| Comparison | N | Figure | Parameter | Statistical tests and significance |
|  |  |  |  |  |
| Controls vs Removing extracellular Ca2+ (*) | 6 , 6 | Fig.3B | Time points of treatment / Min | Two-tailed Student's t-test; p= |
|  |  |  | 0 | 0.357 |
|  |  |  | 25 | 0.641 |
|  |  |  | 50 | 0.498 |
|  |  |  | 75 | 0.108 |
|  |  |  | 100 | 0.069 |
|  |  |  | 125 | 0.048 |
|  |  |  | 150 | 0.014 |
|  |  |  | 175 | 0.005 |
|  |  |  | 200 | 0.002 |
|  |  |  | 225 | 0.002 |
|  |  |  | 250 | 0.002 |
|  |  |  | 275 | 0.002 |
|  |  |  | 300 | 0.001 |
|  |  |  |  |  |
| Removing extracellular Ca2+ , max effect vs maintenance (+) | 6 , 6 | Fig.3B | Time points of treatment / Min | Paired Two-tailed Student's t-test; p= |
|  |  |  | 100 vs 125 | 0.321 |
|  |  |  | 100 vs 150 | 0.038 |
|  |  |  | 100 vs 175 | 0.003 |
|  |  |  | 100 vs 200 | 0.0009 |
|  |  |  | 100 vs 225 | 0.0005 |
|  |  |  | 100 vs 250 | 0.0008 |
|  |  |  | 100 vs 275 | 0.0001 |
|  |  |  | 100 vs 300 | 0.00006 |
|  |  |  |  |  |
|  |  |  | Time points of treatment / Min | Two-tailed Student's t-test; p= |
| Controls vs Removing intra- and extracellular Ca2+ together (*) | 6 , 6 | Fig.3D | 0 | 0.845 |
|  |  |  | 25 | 0.69 |
|  |  |  | 50 | 0.15 |
|  |  |  | 75 | 0.116 |
|  |  |  | 100 | 0.515 |
|  |  |  | 125 | 0.877 |
|  |  |  | 150 | 0.444 |
|  |  |  | 175 | 0.324 |
|  |  |  | 200 | 0.217 |
|  |  |  | 225 | 0.19 |
|  |  |  | 250 | 0.107 |
|  |  |  | 275 | 0.051 |
|  |  |  | 300 | 0.018 |
|  |  |  |  |  |
|  |  |  | Time points of treatment / Min | Paired Two-tailed Student's t-test; p= |
| Removing intra- and extracellular Ca2+ together, max effect vs maintenance (+) | 6 , 6 | Fig.3D | 100 vs 125 | 0.004 |
|  |  |  | 100 vs 150 | 0.001 |
|  |  |  | 100 vs 175 | 0.001 |
|  |  |  | 100 vs 200 | 0.0008 |
|  |  |  | 100 vs 225 | 0.001 |
|  |  |  | 100 vs 250 | 0.004 |
|  |  |  | 100 vs 275 | 0.002 |
|  |  |  | 100 vs 300 | 0.004 |
|  |  |  |  |  |
|  |  |  | Time points of treatment / Min | Two-tailed Student's t-test; p= |
| Controls vs 1µM TTX (*) | 6 , 5 | Fig.3F | 0 | 0.995 |
|  |  |  | 25 | 0.286 |
|  |  |  | 50 | 0.463 |
|  |  |  | 75 | 0.931 |
|  |  |  | 100 | 0.298 |
|  |  |  | 125 | 0.053 |
|  |  |  | 150 | 0.045 |
|  |  |  | 175 | 0.096 |
|  |  |  | 200 | 0.11 |
|  |  |  | 225 | 0.078 |
|  |  |  | 250 | 0.097 |
|  |  |  | 275 | 0.08 |
|  |  |  | 300 | 0.043 |
|  |  |  |  |  |
|  |  |  | Time points of treatment / Min | Paired Two-tailed Student's t-test; p= |
| 1µM TTX, max effect vs maintenance (+) | 6 , 5 | Fig.3F | 100 vs 125 | 0.174 |
|  |  |  | 100 vs 150 | 0.129 |
|  |  |  | 100 vs 175 | 0.279 |
|  |  |  | 100 vs 200 | 0.244 |
|  |  |  | 100 vs 225 | 0.014 |
|  |  |  | 100 vs 250 | 0.065 |
|  |  |  | 100 vs 275 | 0.07 |
|  |  |  | 100 vs 300 | 0.025 |
|  |  |  |  |  |
|  |  |  | Time points of treatment / Min | Two-tailed Student's t-test; p= |
| Controls vs 0.5% procaine, initiation (blue *) | 6 , 5 | Fig.3H | 0 | 0.765 |
|  |  |  | 25 | 0.98 |
|  |  |  | 50 | 0.068 |
|  |  |  | 75 | 0.003 |
|  |  |  | 100 | 0.001 |
|  |  |  | 125 | 0.001 |
|  |  |  | 150 | 0.002 |
|  |  |  | 175 | 0.002 |
|  |  |  | 200 | 0.003 |
|  |  |  | 225 | 0.005 |
|  |  |  | 250 | 0.006 |
|  |  |  | 275 | 0.007 |
|  |  |  | 300 | 0.005 |
|  |  |  |  |  |
|  |  |  | Time points of treatment / Min | Two-tailed Student's t-test; p= |
| Controls vs 0.5% procaine, maintenance (pink *) | 6 , 6 | Fig.3H | 0 | 0.995 |
|  |  |  | 25 | 0.168 |
|  |  |  | 50 | 0.333 |
|  |  |  | 75 | 0.935 |
|  |  |  | 100 | 0.266 |
|  |  |  | 125 | 0.035 |
|  |  |  | 150 | 0.009 |
|  |  |  | 175 | 0.009 |
|  |  |  | 200 | 0.01 |
|  |  |  | 225 | 0.011 |
|  |  |  | 250 | 0.012 |
|  |  |  | 275 | 0.013 |
|  |  |  | 300 | 0.01 |
|  |  |  |  |  |
|  |  |  | Time points of treatment / Min | Paired Two-tailed Student's t-test; p= |
| 0.5% procaine maintenance, max effect vs maintenance (+) | 6 , 6 | Fig.3H | 100 vs 125 | 0.049 |
|  |  |  | 100 vs 150 | 0.004 |
|  |  |  | 100 vs 175 | 0.0005 |
|  |  |  | 100 vs 200 | 0.002 |
|  |  |  | 100 vs 225 | 0.004 |
|  |  |  | 100 vs 250 | 0.006 |
|  |  |  | 100 vs 275 | 0.004 |
|  |  |  | 100 vs 300 | 0.0005 |
|  |  |  |  |  |
|  |  |  | Time points of treatment / Min | Two-tailed Student's t-test; p= |
| Controls vs 0.05% sodium azide, initiation (blue *) | 6 , 6 | Fig.3J | 0 | 0.215 |
|  |  |  | 25 | 0.974 |
|  |  |  | 50 | 0.383 |
|  |  |  | 75 | 0.015 |
|  |  |  | 100 | 0.005 |
|  |  |  | 125 | 0.009 |
|  |  |  | 150 | 0.015 |
|  |  |  | 175 | 0.031 |
|  |  |  | 200 | 0.066 |
|  |  |  | 225 | 0.13 |
|  |  |  | 250 | 0.21 |
|  |  |  | 275 | 0.332 |
|  |  |  | 300 | 0.392 |
|  |  |  |  |  |
|  |  |  | Time points of treatment / Min | Two-tailed Student's t-test; p= |
| Controls vs 0.05% sodium azide, maintenance (pink *) | 6 , 6 | Fig.3J | 0 | 0.569 |
|  |  |  | 25 | 0.005 |
|  |  |  | 50 | 0.052 |
|  |  |  | 75 | 0.234 |
|  |  |  | 100 | 0.436 |
|  |  |  | 125 | 0.835 |
|  |  |  | 150 | 0.181 |
|  |  |  | 175 | 0.072 |
|  |  |  | 200 | 0.095 |
|  |  |  | 225 | 0.193 |
|  |  |  | 250 | 0.426 |
|  |  |  | 275 | 0.782 |
|  |  |  | 300 | 0.621 |
|  |  |  |  |  |
|  |  |  | Time points of treatment / Min | Paired Two-tailed Student's t-test; p= |
| 0.05% sodium azide maintenance, max effect vs maintenance (+) | 6 , 6 | Fig.3J | 100 vs 125 | 0.189 |
|  |  |  | 100 vs 150 | 0.007 |
|  |  |  | 100 vs 175 | 0.002 |
|  |  |  | 100 vs 200 | 0.003 |
|  |  |  | 100 vs 225 | 0.008 |
|  |  |  | 100 vs 250 | 0.132 |
|  |  |  | 100 vs 275 | 0.514 |
|  |  |  | 100 vs 300 | 0.757 |
|  |  |  |  |  |
|  |  |  |  |  |
| Fig. 3 |  |  | Parameter | Statistical tests and significance |
| Comparison | N | Figure |  |  |
|  |  |  | Time points of treatment / Min | Paired Two-tailed Student's t-test; p= |
| Corpus callosum vs Cerebral cortex | 7, 7 | Fig.4I | 0 | 0.751 |
|  |  |  | 100 | 0.00002 |
|  |  |  | 200 | 0.000004 |
|  |  |  | 300 | 0.000007 |
|  |  |  | 400 | 0.000007 |
|  |  |  | 500 | 0.000007 |
|  |  |  |  |  |
|  |  |  | Time points of treatment / Min | Paired Two-tailed Student's t-test; p= |
| Corpus callosum, after OA vs before OA (pink +) | 7, 7 | Fig.4I | 400 vs 300 | 0.083 |
|  |  |  | 500 vs 300 | 0.002 |
|  |  |  |  |  |
|  |  |  |  |  |
|  |  |  | Time points of treatment / Min | Paired Two-tailed Student's t-test; p= |
| Thalamus vs Cerebral cortex | 7, 7 | Fig.4I | 0 | 0.936 |
|  |  |  | 100 | 0.004 |
|  |  |  | 200 | 0.0005 |
|  |  |  | 300 | 0.0002 |
|  |  |  | 400 | 0.0003 |
|  |  |  | 500 | 0.0003 |
|  |  |  |  |  |
|  |  |  | Time points of treatment / Min | Paired Two-tailed Student's t-test; p= |
| Thalamus, after OA vs before OA (blue +) | 7, 7 | Fig.4I | 400 vs 300 | 0.488 |
|  |  |  | 500 vs 300 | 0.003 |
|  |  |  |  |  |
